# Prevalence and risk factors of refractive error in Qinghai, China: a cross-sectional study in Han and Tibetan adults in Xining and surrounding areas

**DOI:** 10.1186/s12886-021-01996-2

**Published:** 2021-06-19

**Authors:** Meng Wang, Linyang Gan, Jiantao Cui, Guangliang Shan, Ting Chen, Xianghua Wang, Yuhan Wang, Li Pan, Zhanquan Li, Sen Cui, Airong Yang, Wenfang Li, Guoqiang Jia, Ximing Han, Huijing He, Yong Zhong, Jin Ma

**Affiliations:** 1 Department of Ophthalmology, Peking Union Medical College Hospital, Chinese Academy of Medical Sciences & Peking Union Medical College, No. 1 Shuaifu Yuan, Dongcheng District, Beijing, 100730 China; 2 Department of Epidemiology and Statistics, Institute of Basic Medical Sciences, Chinese Academy of Medical Sciences & Peking Union Medical College, Beijing, China; 3 Qinghai University Affiliated Hospital, Xining City, Qinghai China

**Keywords:** Refractive error, Risk factors, Adults, Han population, Tibetan population

## Abstract

**Background:**

Our study aimed to explore the prevalence and risk factors of refractive error (RE) in Han and Tibetan population aged 50–79 years in Xining and surrounding areas in Qinghai Province on Qinghai-Tibet Plateau.

**Methods:**

As part of the China National Health Survey, our cross-sectional study compared the age-adjusted prevalence of RE in Han and Tibetan older adults aged 50–79 years in Xining and surrounding areas. A multivariate logistic regression model was used to identify risk factors for myopia and hyperopia.

**Results:**

Among 769 Han participants and 476 Tibetan participants, the age-adjusted prevalence of myopia (spherical equivalent (SE) < − 0.5D), hyperopia (SE > + 0.5D), high myopia (SE < -6.0D) and astigmatism (cylindrical equivalent > = 0.5D) is 28.56, 22.82, 2.80, and 69.38%. Han participants have higher age-adjusted prevalence of myopia (32.93% vs 21.64%, *p* < 0.001), high myopia (3.93% vs 1.02%, *p* = 0.001) and astigmatism (72.14% vs 64.94%, *p* = 0.021) compared to Tibetan participants. Being Tibetan is the protective factor of myopia compared to being Han (OR 0.58, 95%CI 0.42–0.79, *p* < 0.001). Older age (*p* = 0.032), longer time length in rural area (*p* = 0.048), undergraduate/graduate education level (*p* = 0.031), lighter active level (*p* = 0.007) and lower BMI (*p* = 0.015) are risk factors for myopia. Older age (all *p* < 0.001) and pterygium status of the same eye (*p* = 0.013) also increase the hyperopia risk.

**Conclusions:**

Our study found an overall prevalence of myopia of 28.56% in Xining and surrounding areas in adults older than 50 years. Han population has higher myopia risk than Tibetan population. More medical and social resources should be allocated to improve the vision and life quality of older adults.

## Background

Refractive error (RE) affects billions of people and uncorrected RE is the most important cause of the visual impairment worldwide [1]. RE could decrease life quality massively and impose a heavy social and economic burden [2]. Higher prevalence of myopia was reported in countries of East Asia including China, Japan and Korea compared to that in other regions. Social and economic factors are closely associated with the incidence of myopia [3]. The Hisayama Study in Japan noticed the significant increase of the myopia prevalence in older adults in Hisayama, from 37.7% in 2005 to 45.8% in 2017 (p for trend < 0.001). Moreover, the prevalence of high myopia increased from 5.8% in 2005 to 9.5% in 2017 [4]. Similarly, the Chinese Eye Study in Singapore also pointed out that the prevalence of myopia and high myopia increases dramatically in older adults compared to that in Tanjong Pagar Survey 12 years earlier [5]. Considering the increasing trend of myopia in the population of the elderly, we should pay attention to the refractive condition in older Chinese in China as well. Additionally, myopia, particularly high myopia, could significantly increase the risk of complications including myopic maculopathy [6], retinal detachment and open-angle glaucoma [7], of which the risk is even higher in older adults [8]. According to the most recent international population report, the distribution of people older than 65 years would increase from 8.5% (617 million) in 2015 to 16.7% (1600 million) in 2050 [9]. More attention should be paid to improve the medical care and health conditions of the older adults. Limited epidemiological researches were about the prevalence of RE in Chinese older adults and most of them focused on eastern regions. The prevalence of myopia of the elderly in China varies from 9.5% (Southern Harbin Eye Study, 2009, *n* = 4979, age ≥ 50y) [10] to 32.3% (Liwan Eye Study, 2009, *n* = 1269, age ≥ 50y) [11].

Located on the Qinghai-Tibet Plateau, Qinghai is one of the widest provinces in China with average altitude higher than 3000 meters [12]. Tibetan is one of the largest ethnic minorities in China, mainly living on Qinghai-Tibet Plateau [13]. Before the Chinese Economic Reform (1978), Tibetan population was less influenced by Eastern China politically and economically, and kept living in pastoral settings and small farming communities due to its remote and plateau location [13]. In 1999, Dunzhu S et al. reported a prevalence of blindness and severe vision loss of Tibetans in Tibet in adults aged over 50 years that is twice or three times higher than that in other Chinese areas [14]. Considering that RE rarely causes blindness, this study did not further investigate the prevalence of RE in Tibet. In 2017, higher prevalence of blindness was again confirmed in Tibetan population compared to Han population (2.2% vs 0.6% p<0.05) in Kandze Tibetan Autonomous Prefecture in Sichuan Province [15]. However, the specific prevalence and risk factors of RE were not investigated. Although Tibetan population has heavy burden of visual impairment, up to now, no epidemiological study has investigated the prevalence of RE in adults in Qinghai, where Tibetan people mainly settle in. There are also plenty of Han people living in Qinghai Province, making it an ideal place to investigate the differences of prevalence and risk factors of RE between Han and Tibetan people.

Our study is part of the China National Health Survey conducted by Chinese Academy of Medical Science. As the capital city of Qinghai Province, Xining is located on the plateau with the average altitude of 2261 m [12]. By investigating the prevalence and risk factors of RE in Tibetan and Han adults older than 50 years in Xining and surrounding areas, our study aimed to provide epidemiological data of Tibetan and Han older adults in plateau areas.

## Methods

### Study population

To evaluate the physiological and health condition of the Chinese, the Chinese Academy of Medical Sciences conducted the China National Health Survey (CNHS) [16]. The part reported in this study was conducted in Qinghai Province in 2015. According to the level of urbanization, six locations were randomly chosen for sampling in this cross-sectional study, including two in a large city (Teaching hospital of Qinghai University and the Revenue Agency of Chengxi District in Xining), one in a medium city (Qiabuqia, the capital of Hainan State), two county seats (the County Hospitals of Guide County and Hualong County), and one relatively less developed location (the Town of Jiangxigou in Gonghe County). All these locations are within 160 km from Xining City. This study was conducted according to the tenets of the Declaration of Helsinki. The ethics approval was received from the bioethical committee of the Institute of Basic Medical Sciences, the Chinese Academy of Medical Sciences. After full explanation of the nature and possible consequences of the study, every participant gave their written informed consent.

### Inclusive and exclusive criteria

Cluster sampling method was used in China National Health Survey. The ratio of participants of Han and Tibetan in our study is similar to the natural proportion of the population in Qinghai Province. As part of the National Health Survey, our study included 1417 participants aged 50–79 years old, and 1245 of them completed a questionnaire, a routine physical examination and eye examination (response rate: 87.9%). Only people who had lived in their current residence for more than 1 year were recruited and psychiatric patients were excluded. Only participants with both Han parents or Tibetan parents were included in our study. Participants who have experienced cataract or myopia surgery were excluded.

### Data collection

#### Questionnaire and routine physical examination

During a comprehensive interview by our well-trained interviewers, a questionnaire about demographic information and health history was collected. Information about age, sex, ethnicity of the participants and their parents, birthplace, current residence, migration date, education level, occupation, income per month, smoking and drinking practice, occupational and leisure-time physical activity, and medical history including hypertension and diabetes status was included in our questionnaire. An assessment of height, weight, blood pressure, and fasting blood-glucose was included in routine physical examinations. Height was measured to the nearest 0.1 cm using a fixed stadiometer and in a standing position by bioelectrical impendence analysis with a commercially available body composition analyzer (BC-420, TANITA, Japan) with participants in light clothes. Body mass index (BMI) was calculated using the formula weight (kg)/height (m)^2^. A digital automatic blood pressure monitor (Omron HEM-907, Japan) was used after resting 10 min and the average of three measurements was recorded to measure systolic and diastolic blood pressure. Blood samples were drawn after fasting overnight for at least 8 h, and then immediately processed, refrigerated, transferred and assessed in the lab in the General Hospital of Chinese Peoples’ Liberation Army, Beijing. Fasting blood glucose (mmol/L) was tested with a chemistry analyzer (ROCHE Cobas8000C701, USA). Hypertension would be defined with either positive hypertension history in the questionnaire or above-normal blood pressure during field investigation. Similarly, diabetes would be defined with either positive diabetes history or above-normal fasting blood glucose we measured.

#### Eye examination

Eye examinations on our participants were performed by well-trained ophthalmologists. A logarithm of the minimum angle of resolution E chart (Wehen Co., Ltd., Guangzhou, China) was used for visual acuity measurement at 4 m. An auto refractor (ARK-510A, Nidek Co., Ltd., Tokyo, Japan) was used to measure noncycloplegic refraction and corneal curvature radium, and the average of three measurements was recorded. The anterior segment of eyes was examined with a portable hand-held slit-lamp (KJ5S2, Suzhou Kangjie Medical Co. Ltd., Jiangsu, China).

#### Stratification standard

All participants were divided into 4 age groups, which were 50–54, 55–59, 60–64, 65 and over years old. Education level was divided into three groups including primary school and lower, middle/high school, and undergraduate/graduate. Occupation information was divided into close-workers (including officers, technicians and workers) and non close-workers (including farmers, waiters, and housewives). Participants were divided into never-smokers and ever-smokers (including current smokers and former smokers) according to tobacco consumption. Additionally, participants were divided into never-drinkers and ever-drinkers (including current drinkers and former drinkers). Occupational physical activity was classified into three categories as light, moderate or heavy according to intensity. Leisure-time physical activity was classified into three levels, according to days of exercise (participation in moderate or vigorous activity for 20 min or more per day) weekly. We merged occupational and leisure-time physical activity into active level and regrouped it into light, moderate and heavy active level [17]. Birthplace and current residence were classified into urban and rural areas, and time length in rural areas was calculated according to the birthplace, current residence and migration date.

### Definitions of RE

In our study, myopia was defined as spherical equivalent (SE) < − 0.5D [18], and hyperopia was defined as SE > + 0.5D. High myopia was defined as SE < -6.0D [18]. Astigmatism was defined as > = 0.5D of the cylinder.

### Statistics

High correlation between right and left eyes was found in our study (Spearman correlation test, *p* < 0.001, r = 0.824). We concluded similar statistical analysis results between right and left eyes and only reported results of the right eye for concision. Chi-square test and t test were used to compare the demographic differences between Han and Tibetan participants. The difference of RE distributions between ethnicities among different age groups was tested with a chi-square test. The risk factors for RE were identified with multivariate logistic regression analysis. The age-standardized prevalence was based on the Sixth National Population Census of the People’s Republic of China. A *p* value less than 0.05 was considered significant. Statistical analysis was done using Stata version 15.1, and figures were created using GraphPad Prism 7.0a.

## Results

### Characteristics of Han and Tibetan adults

One thousand two hundered forty-five participants aged 50–80 years old, including 769 Han participants and 476 Tibetan participants were included in the final analysis (Table 1). The age composition is similar between Han and Tibetan population (*p* = 0.658). No difference was found in cigarette consumption (*p* = 0.127), hypertension (*p* = 0.065) and pterygium (*p* = 0.395) between Han and Tibetan population. However, Tibetan population has heavier weight and higher BMI compared to Han population (all *p* < 0.001). More participants of Tibetan ethnicity were born and now live in rural area and therefore have longer time length spent in rural area (all p < 0.001). Moreover, more Han participants are influenced by diabetes (13.13% vs 8.61%, *p* = 0.015), and are with moderate and heavy active level (light: 11.18% vs 17.23%; moderate: 73.86% vs 70.80%; heavy: 14.95% vs 11.97%; *p* = 0.006).

**Table 1:** Characteristics of Han and Tibetan participants

	Han ***n*** = 769	%	Tibetan ***n*** = 476	%	***P*** value
**Sex**					0.035
**Male**	376	48.89	262	55.04	
**Female**	393	51.11	214	44.96	
**Age (y)**					0.658
**50–54**	359	46.68	207	43.49	
**55–59**	145	18.86	96	20.17	
**60–65**	136	17.69	94	19.75	
**65+**	129	16.78	79	16.60	
**Height (cm)**	162.37		163.00		0.205
**Weight (kg)**	63.99		68.34		<0.001
**BMI (kg/m**^**2**^**)**	24.22		25.62		< 0.001
**Current Residence**					<0.001
**Urban**	528	68.66	200	42.02	
**Rural**	241	31.34	276	57.98	
**Birth Place**					<0.001
**Urban**	167	21.72	28	5.88	
**Rural**	602	78.28	448	94.12	
**Time spent in rural areas (y)**	33.48		47.48		<0.001
**Education**					<0.001
**Primary school or lower**	327	42.52	355	74.58	
**Middle/high school**	292	37.97	74	15.55	
**Undergraduate/graduate**	150	19.51	47	9.87	
**Occupation**					<0.001
**Non-close**	420	54.62	361	75.84	
**Close**	349	45.38	115	24.16	
**Income (¥)**					<0.001
**< 800/month**	273	35.50	285	59.87	
**800–2000/month**	165	21.46	88	18.49	
**> 2000/month**	331	43.04	103	21.64	
**Hypertension**	315	40.96	170	35.71	0.065
**Diabetes**	101	13.13	41	8.61	0.015
**Smoking**					0.127
**Never**	471	61.25	312	65.55	
**Past/current**	298	38.75	164	34.45	
**Drinking**					<0.001
**Never**	360	46.81	291	61.13	
**Past/current**	409	53.19	185	38.87	
**Active level**					0.006
**Light**	86	11.18	82	17.23	
**Moderate**	568	73.86	337	70.80	
**Heavy**	115	14.95	57	11.97	
**Pterygium**	59	7.67	43	9.03	0.395

### Prevalence of RE in Han and Tibetan population

The age-adjusted prevalence of myopia, hyperopia, high myopia and astigmatism in our study is 28.56% (95%CI 25.77–31.35%), 22.82% (95%CI 20.13–25.52%), 2.80% (95%CI 1.85–3.74%), and 69.38% (95%CI 66.71–72.06%), as presented in Table 2. Higher age-adjusted prevalence of myopia (32.93% vs 21.64%, *p* < 0.001), high myopia (3.93% vs 1.02%, *p* = 0.001) and astigmatism (72.14% vs 64.94%, *p* = 0.021) was found in Han population compared to Tibetan population. The age-adjusted prevalence of hyperopia is similar between Han and Tibetan participants (23.04% vs 22.48%, *p* = 0.313).

**Table 2:** Crude and age-adjusted prevalence of refractive error

	N	Crude rate	Age-adjusted rate	95%CI lower	95%CI upper	*P* value
**Myopia**						
**Total**	373	29.96%	28.56%	25.77%	31.35%	<0.001
**Han**	274	35.63%	32.93%	29.28%	36.58%	
**Tibetan**	99	20.80%	21.64%	17.46%	25.82%	
**Hyperopia**						
**Total**	228	18.31%	22.82%	20.13%	25.52%	
**Han**	136	17.69%	23.04%	19.57%	26.51%	0.313
**Tibetan**	92	19.33%	22.48%	18.20%	26.75%	
**High myopia**						
**Total**	42	3.37%	2.80%	1.85%	3.74%	
**Han**	36	4.68%	3.93%	2.51%	5.35%	0.001
**Tibetan**	6	1.26%	1.02%	0.06%	1.98%	
**Astigmatism**						
**Total**	803	64.50%	69.38%	66.71%	72.06%	
**Han**	515	66.97%	72.14%	68.87%	75.40%	0.021
**Tibetan**	288	60.50%	64.94%	60.38%	69.49%	

### Distribution of RE and SE in different age groups

**Fig. 1:**
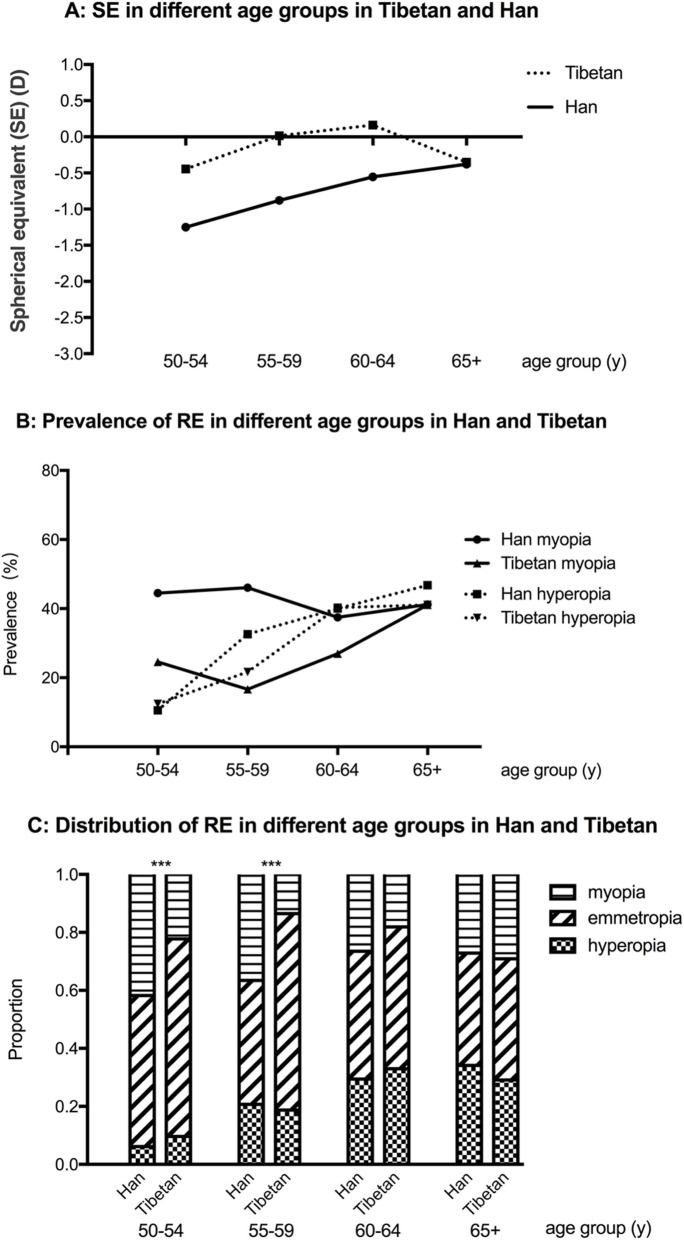
SE indifferent age groups in Tibetan and Han (**a**); Prevalence of RE in different age groups in Han and Tibetan (**b**) and Distribution of RE in different age groups in Han and Tibetan (**c**), *** *p* < 0.001

Participants were divided into four age groups including 50–54, 55–59, 60–64, and 65 and over years old. Less myopic mean SE was found in Han population with older age (Fig. 1a). The prevalence of hyperopia is also higher in older age group in both Han and Tibetan participants (Fig. 1b). The U-shaped curve was noticed in the prevalence of myopia in different age groups in both Han and Tibetan population (Fig. 1b). Difference was found in the distribution of myopia, hyperopia and emmetropia in 50–54 and 55–59 age groups between Han and Tibetan population (50–54 *p* < 0.001, 55–59 p < 0.001) (Fig. 1c).

### Risk factors of myopia and hyperopia

**Fig. 2:**
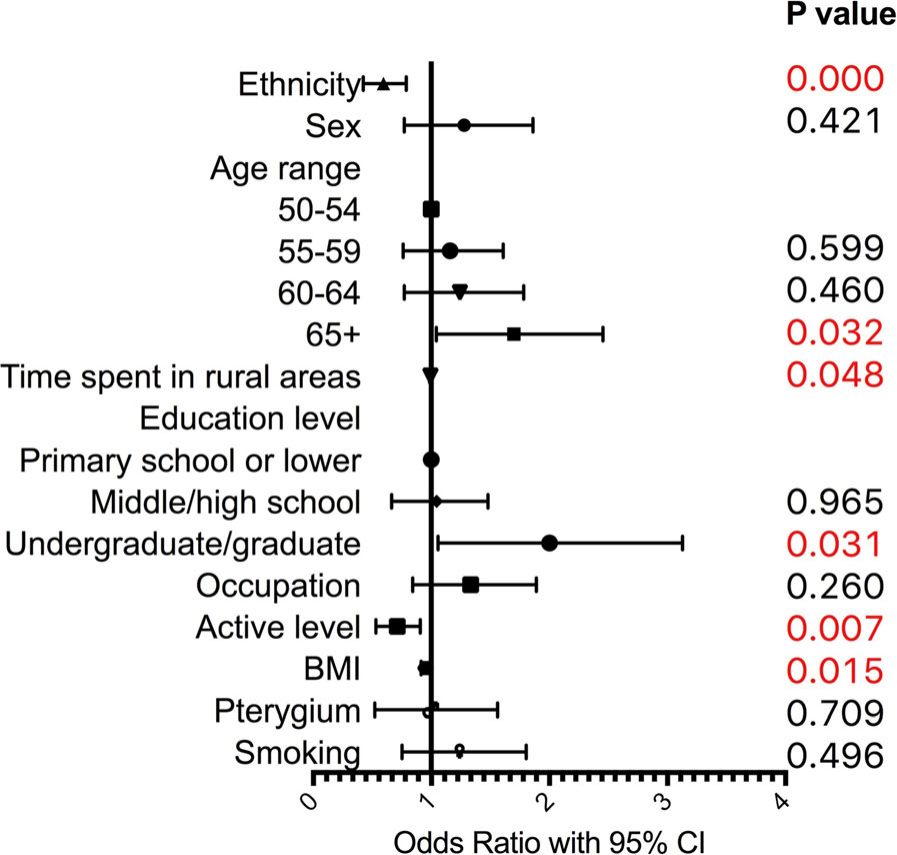
Results of multivariate logistic regression of myopia in Han and Tibetan populations

**Fig. 3:**
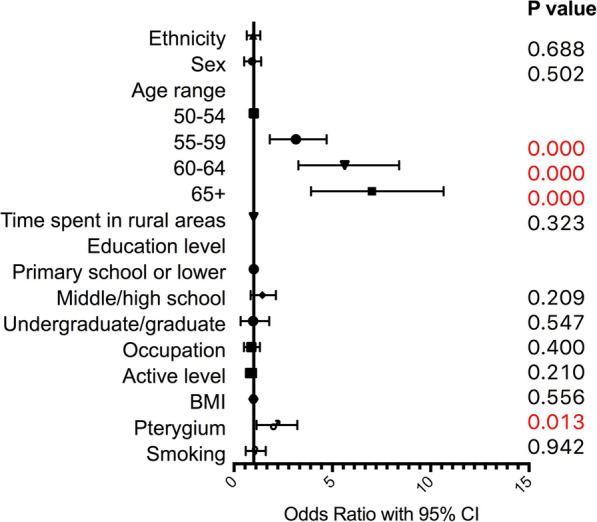
Results of multivariate logistic regression of hyperopia in Han and Tibetan populations

A multivariate logistic regression with 10 indicators was used to identify risk factors of myopia and hyperopia and results were presented in Table 3, Figs. 2 and 3. Ethnicity affects the risk of myopia and Tibetan population has less risk of myopia compared to Han population (OR 0.58, 95%CI 0.42–0.79, p < 0.001). However, ethnicity is not the risk of hyperopia in Han population compared to Tibetan population (OR 0.93, 95%CI 0.65–1.33, *p* = 0.688).

**Table 3:** Results of multivariate logistic regression of refractive errors in Han and Tibetan

	Myopia			Hyperopia					High myopia				Astigmatism			
	Odds Ratio	95%CI lower	95%CI upper	***P*** value	Odds Ratio	95%CI lower	95%CI upper	***P*** value	Odds Ratio	95%CI lower	95%CI upper	***P*** value	Odds Ratio	95%CI lower	95%CI upper	***P*** value
**Ethnicity**	0.58	0.42	0.79	0.000	0.93	0.65	1.33	0.688	0.40	0.15	1.10	0.077	0.78	0.59	1.02	0.070
**Sex**	1.20	0.77	1.86	0.421	0.85	0.52	1.38	0.502	1.88	0.60	5.90	0.279	1.22	0.85	1.76	0.278
**Age period**																
**50–54**	1.00				1.00				1.00				1.00			
**55–59**	1.11	0.76	1.61	0.599	2.92	1.81	4.71	0.000	0.53	0.18	1.59	0.260	1.62	1.17	2.25	0.004
**60–64**	1.17	0.77	1.78	0.460	5.24	3.27	8.39	0.000	0.62	0.20	1.95	0.415	2.29	1.60	3.28	0.000
**65+**	1.60	1.04	2.45	0.032	6.46	3.92	10.65	0.000	0.53	0.15	1.86	0.322	4.29	2.80	6.57	0.000
**Time in rural area**	0.99	0.99	1.00	0.048	1.00	0.99	1.00	0.323	0.99	0.97	1.01	0.510	0.99	0.99	1.00	0.045
**Education**																
**< =primary school**	1.00				1.00				1.00				1.00			
**middle/High school**	0.99	0.66	1.48	0.965	1.34	0.85	2.13	0.209	0.34	0.10	1.15	0.082	1.01	0.71	1.44	0.967
**undergraduate/graduate**	1.82	1.06	3.13	0.031	0.77	0.33	1.79	0.547	0.57	0.14	2.30	0.431	1.05	0.63	1.73	0.857
**Occupation**	1.26	0.84	1.89	0.260	0.81	0.50	1.32	0.400	1.63	0.51	5.24	0.414	0.79	0.55	1.15	0.220
**Active level**	0.69	0.53	0.91	0.007	0.82	0.60	1.12	0.210	1.03	0.45	2.32	0.950	0.89	0.71	1.13	0.347
**BMI**	0.95	0.91	0.99	0.015	0.99	0.94	1.03	0.556	1.01	0.91	1.12	0.820	1.00	0.97	1.04	0.924
**Pterygium**	0.90	0.52	1.56	0.709	1.92	1.15	3.22	0.013	0.26	0.03	2.14	0.210	1.50	0.93	2.41	0.097
**Smoking**	1.16	0.75	1.80	0.496	0.98	0.60	1.61	0.942	0.41	0.11	1.53	0.187	1.33	0.92	1.93	0.127

*Note*: Multivariate logistic regression model with 10 indicators including ethnicity, sex, age period, rural level, education level, occupation, active level, BMI, pterygium status of the same eye, and smoking practice was used to assess risk factors for myopia, hyperopia, high myopia and astigmatism. Myopia and hyperopia results were compared to emmetropic individuals. High myopia results were compared to individuals with light or moderate myopia. OR of ethnicity was Tibetan/Han. OR of sex was female/male. OR of occupation was close-workers/non close-workers. OR of Pterygium was the eye with pterygium/eye without pterygium. OR of smoking was individual smoked/individual never smoked

Compared to participants aged 50–54 years, those aged 65+ (OR 1.60, 95%CI 1.04–2.45, *p* = 0.032) years have higher risk of myopia. Longer time length in rural area is a protective factor for myopia (*p* = 0.048). Compared to those with primary school or lower education level, those with undergraduate or graduate education level have much higher risk to be influenced by myopia (OR 1.82, 95%CI 1.06–3.13, *p* = 0.031). Myopia is also associated to active level (OR 0.69, 95%CI 0.53–0.91, *p* = 0.007) and BMI (OR 0.95, 95%CI 0.91–0.99, *p* = 0.015).

Age is notably related to hyperopia. Compared to those who aged 50–54 years, older participants who aged 55–59, 60–64, 65+ (all *p* < 0.001) years have much higher risk of hyperopia. Older group have higher OR and the OR for age group 65+ years reaches 6.46 (95%CI 3.92–10.65). Moreover, the pterygium status of the same eye also increases the hyperopia risk (OR 1.92, 95%CI 1.15–3.22, *p* = 0.013).

### Spectacle coverage rate of myopia

Among 373 participants of myopia in both Han and Tibetan in our study, only 67 of them wear glasses (17.96%). There are 274 Han participants affected by myopia, and 58 of them wear glasses (21.17%). However, only 9 of 99 Tibetan myopia participants wear glasses (9.09%). The spectacle coverage rate of myopia in Han is particularly higher than that in Tibetan (*p* = 0.007).

## Discussion

### Myopia prevalence in older adults of Han population in Qinghai and other areas

Our study is the first study investigating RE prevalence in older adults in Qinghai. The age-adjusted myopia prevalence is as high as 28.56% in Xining and surrounding areas in Han and Tibetan population of 50–79 years old. The myopia prevalence in Han population is significantly higher than that in Tibetan population (32.93% vs 21.64%). Interestingly, studies in more developed areas in Eastern China and Southeastern Asia focusing on Han population found similar prevalence of myopia (Table 4). Up to now, there are limited number of epidemiological studies about RE in older adults in China [10, 11, 18–24]. The highest prevalence of myopia was observed in Liwan Eye Study in 2009, 32.3% in 1269 adults older than 50 years [11], while the prevalence of myopia in Han population in our study is 32.93%. Although it is hard to compare the myopia prevalence among studies with massive variety in the age distribution of the participants and urban and rural settings, the prevalence of myopia we found in Han population in Xining and surrounding areas is not low.

**Table 4:** Comparison of reported prevalence of RE in selected population-based studies nationally and internationally

Studies	n	Population	Age (y)	Myopia (%)	Myopia SE	High myopia (%)	High myopia SE	Hyperopia (%)	Hyperopia SE
**CNHS Inner Mongolia Study, 2019** [19]	2090	Chinese (Han/Mongolian)	40–80	29.4	<−0.5D	3.6	<−6D	28.4	> 0.5D
**CNHS Yunnan Study, 2019** [20]	1626	Chinese (Han/Yi)	40–80	26.4	<− 0.5D	2.6	<−6D	19.9	> 0.5D
**Handan Eye Study, 2009** [21]	6491	Chinese	30–86	26.7	<−0.5D	1.8%	<−5D	15.9	> 0.5D
**Beijing Eye Study, 2006** [18]	4319	Chinese	40–90	22.9	<−0.5D	2.6	<−6D	NR	NR
**Liwan Eye Study, 2009** [11]	1269	Chinese	≥50	32.3	<−0.5D	5.0	<−5D	40.0	> 0.5D
**Shihpai Eye Study, 2003** [22]	1361	Chinese	≥65	19.4	<−0.5D	2.4	<−6D	59.0	> 0.5D
**Shanghai Eye Study, 2017** [23]	6099	Chinese	≥50	22.8	<−0.5D	4.6	<−6D	48.5	> 0.5D
**Suzhou Rural Eye Study, 2017** [24]	4795	Chinese	≥60	23.5	<−0.5D	2.1	<−6D	NR	NR
**Rural Southern Harbin Eye Study, 2009** [10]	4979	Chinese	≥50	9.5	<−0.5D	NR	NR	8.9	> 0.5D
**Tanjong Pagar Study, 2000** [25]	1232	Chinese (Singapore)	40–79	38.7	<−0.5D	9.1	<−5D	28.4	> 0.5D
**Singapore Longitudinal Aging Study, 2011** [26]	1727	Chinese (Singapore)	55–85	30.8	≤ − 0.5D	NR	NR	41.1	≥1D
**Chinese American Eye Study, 2017** [27]	4144	Chinese (American)	≥50	35.1	<−0.5D	7.4	<−5D	40.2	> 0.5D
**Tajimi Study, 2008** [28]	3021	Japanese	≥40	41.8	<−0.5D	8.2	<−5D	27.9	> 0.5D
**Hisayama Study, 2019** [4]	2936	Japanese	≥40	45.8	<−0.5D	9.5	<−5D	NR	NR
**Namil Rural Study, 2013** [29]	1215	Korean	≥40	20.5	<−0.5D	1.0	<−6D	41.8	> 0.5D
**Singapore Malay Eye Study, 2008** [30]	2974	Malay (Singapore)	40–79	26.2	<−0.5D	3.9	<−5D	27.4	> 0.5D
**Singapore Indian Eye Study, 2011** [31]	2805	Indian (Singapore)	40–79	28.0	<−0.5D	4.1	<−5D	35.9	> 0.5D
**Andhra Pradesh Eye Disease Study, 2009** [32]	3642	Indian	40–92	34.6	<−0.5D	4.5	<−5D	18.4	> 0.5D
**Indian Study of Age-related Eye Disease, 2018** [33]	3267	Indian	≥40	35.6	≤ − 0.75D	2.0	≤ − 6D	30.3	≥0.5D
**Meiktila eye study, 2008** [34]	1863	Burmese	≥40	42.7	<−1D	6.5	<−6D	15.0	>1D
**Six Villages in Sumatra, 2002** [35]	358	Indonesian	≥40	34.1	≤ − 0.5D	NR	NR	32.1	≥0.5D
**Shahroud Eye Cohort Study, 2012** [36]	4864	Iranian	40–64	30.2	≤ − 0.5D	1.9	<−6D	35.6	> 0.5D
**Mongolian Eye Study, 2004** [37]	1617	Mongolian	≥40	17.2	<−0.5D	NR	NR	49.9	> 0.5D
**National Health and Nutrition Examination Survey, 2008** [38]	7357	American	≥40	31.0	≤ − 1D	6.0	≤ − 5D	5.3	≥3D
**Multi-Ethnic Study of Atherosclerosis, 2013** [39]	4430	American	45–84	25.1	≤ − 1D	4.6	≤ − 5D	38.2	≥1D
**Los Angeles Latino Eye Study, 2006** [40]	5927	Latinos (American)	≥40	16.8	≤ − 1D	2.4	≤ − 5D	NR	NR
**Barbados Eye Study, 1999** [41]	4709	Barbados-born Black adults	40–84	21.9	<−0.5D	NR	NR	46.9	> 0.5D
**Victoria Visual Impairment Project, 1999** [42]	4532	Australian	40–98	17.0	<−0.5D	2.1	<−5D	37.0	> 0.5D

*NR* not reported

Three potential reasons could lead to such similarity in the prevalence of myopia between our study and other studies in more developed areas. Firstly, living on the Qinghai-Tibet Plateau, residents in Xining and surrounding areas experience severer ultraviolet exposure, which could lead to higher occurrence of cataract [43]. The prevalence of age-related cataract in Lhasa, the capital city of Tibet with altitude of 4000 m, is 60% higher than that in plain areas [44]. Higher incidence of cataract due to high altitude could cause the myopia shift and therefore increase the prevalence of myopia. Secondly, the prevalence of myopia increases a lot in older adults recently. A dramatic increase of the prevalence of myopia and high myopia in older adults is noticed both in Japan [4] and Singapore [5]. With the constantly increasing prevalence of myopia in adolescent, it is reasonable to see higher prevalence of myopia in older adults considering the aging of the population. The known prevalence of myopia as references in older adults were collected years ago, which could have already increased nowadays. Thirdly, with the China Western Development Policy, the difference of social and economic environment between Western China and Eastern China, which is closely related to myopia occurrence, is getting much smaller. Moreover, Xining and surrounding areas are most developed region on Qinghai-Tibet Plateau, which might explain the similar prevalence of myopia in our study and in Eastern China. More medical resources should be paid to monitor the vision of local residents in Western China, and medical staff should be cautious of myopic complications considering the comparable prevalence of myopia.

### The impact of ethnicity on the prevalence of RE

The prevalence of myopia, high myopia and astigmatism in Han population is significantly higher than that in Tibetan population. After adjusted by sex, age, time length in rural area, education level, occupation, active level, BMI, pterygium, and smoking condition, the risk of myopia in Han population is considerably higher than that in Tibetan population (*p* < 0.001). Our research group also found that in Inner Mongolia, the myopia risk is significantly higher in Han population than Mongolian population, which is one of the biggest ethnic minorities in China [19]. After investigating 10,333 Chinese, Indian and Malaysian in Singapore, Pan CW found that the prevalence of myopia, high myopia and astigmatism is higher among Chinese than that in Indian and Malaysian [5]. The Chinese population in Singapore is mostly Han population. In our study, Han population has higher risk of myopia than Tibetan. However, considering that there are still many other factors such as economic condition, healthcare access, sleeping time length [12], genetic background and so on, the difference of the myopia risk between Han and Tibetan population might be the results of environmental and genetic variance.

The prevalence of myopia is lower in Tibetan population but the prevalence of the uncorrected myopia is much higher compared to Han population. Uncorrected refractive error is the most important reason of visual impairment worldwide [45]. The prevalence of uncorrected RE is excessive in older population in China, and the prevalence of uncorrected myopia is even higher [46]. Therefore, to improve the visual quality and life quality of older people, government and medical service provider should pay extra attention to the correction of RE in older population.

The result of the multivariate logistic regression did not show any difference between the risk of hyperopia in Han and Tibetan population. The occurrence of hyperopia might be much more influenced by age, and could be similar among different ethnic populations.

### The change of prevalence of myopia and hyperopia with aging

The distribution of myopia, hyperopia and emmetropia varies significantly in Han and Tibetan population aged 50–54 years and 55–59 years. However, in people older than 60 years, no difference was noticed between these two ethnic populations. With aging process, the incidence of cataract and hyperopia increases and the effect of the refractive conditions in younger period of life would be less after 60 years old. Higher odds ratio of hyperopia is found with older age, which is correspond with normal process of aging [11], as well as less education in older generations.

Meanwhile, we noticed the U-shaped curve of the prevalence of myopia in people older than 50 years in both Han and Tibetan population. Such U-shaped curve was also found in Tanjong Pajar study in Singapore [25], Malaysian Eye Study in Singapore [30], Handan Eye Study in China [21], and Sumatra Study in Indonesia [35]. The risk of myopia first decreases with aging but starts to increase after 65 years old. The prevalent nuclear and posterior subcapsular cataract are significantly associated with myopia [47]. Therefore, the association between aging and myopia could be related to the myopic shift due to cataract [47].

### Other risk factors of myopia and hyperopia

Shorter time length in rural area, higher education level, lighter active level and lower BMI were identified as risk factors for myopia in our study. Similar study conducted by our team in Inner Mongolia of China also found that longer time length in rural area decreases the risk of myopia(*p* < 0.001, 19). A study in Beijing with 681 students showed a much lower myopia risk in rural area compared to that under urban settings (OR 0.17, p < 0.001, [48]. Similarly, the risk of myopia in inner city region with higher population concentration was found to exceed that in outer suburban region in another study including 2367 Australian children (17.8% vs 6.9%) [49]. The higher education level is widely considered to be related to higher incidence of myopia, which was proved again in our study. The excessive amount of near vision work might explain the higher risk of myopia in population with higher education level [1, 19, 50, 51]. Terasaki H et al. noticed the association between BMI and axial length [52]. The body structure especially the height is correlated to the axial length [26, 48, 53–55]. Lower BMI is the risk factor of myopia in our study, which might be explained by the influence of BMI on the axial length. Seang-Mei Saw also found that eyes in Singapore Chinese children who had a higher BMI tended to have refractions that were more hyperopic [55]. Moreover, people with higher educated level generally have lower BMI [56], which might also influence the risk of myopia.

Pterygium is another risk factor of hyperopia in our study apart from age, which was also found in the study conducted by our team in Inner Mongolia and Yunnan [19, 20]. Eyes with hyperopia tend to have thinner cornea and sclera, but thicker conjunctiva, which might increase the risk of pterygium [57, 58]. Pterygium would also drag the cornea and make it flatter, which might induce hyperopia. Meanwhile, the association between pterygium and aging [59] might also increase the association between pterygium and hyperopia.

### Limitation

Our study was conducted mainly in Xining, the capital city of Qinghai, and surrounding areas, which cannot show the overall picture of the RE condition in plateau regions. As a cross-sectional study, our study cannot prove the causality between risk factors and RE. Therefore, more longitudinal studies including cohort study and prospective study are highly recommended in the future.

## Conclusions

Located on the remote Qinghai-Tibet Plateau, Qinghai Province is mainly composed of Han and Tibetan population, with no previous epidemiological research about RE in older adults. In our study, an overall prevalence of myopia of 28.56% was found in Xining and surrounding areas in adults older than 50 years. Han population has higher myopia risk than that in Tibetan population. Older age, shorter time length in rural area, lighter active level, and lower BMI are risk factors of myopia. Older age and pterygium increase the risk of hyperopia. The myopia prevalence of older adults in relatively developed regions in Western China was not as low as expected. More medical and social resources should be allocated to improve the vision and life quality of older adults in this area.

## Data Availability

The datasets generated and/or analysed during the current study are not publicly available due to the restriction set by China National Health Survey but are available from the corresponding author on reasonable request.

## Abbreviations

BMI: Body mass index
CNHS: China National Health Survey
CI: Confidence interval
OR: Odds ratio
RE: Refractive error
SE: Spherical equivalent
